# Cerebrospinal fluid levels of neuroinflammatory biomarkers are increased in athletes with persistent post-concussive symptoms following sports-related concussion

**DOI:** 10.1186/s12974-023-02864-0

**Published:** 2023-08-17

**Authors:** Anna Gard, Fredrik Vedung, Fredrik Piehl, Mohsen Khademi, Maria Portonova Wernersson, Ia Rorsman, Yelverton Tegner, Hélène Pessah-Rasmussen, Karsten Ruscher, Niklas Marklund

**Affiliations:** 1 Department of Clinical Sciences Lund, Neurosurgery, Lund University, Lund, Sweden; 2 Department of Neuroscience, Neurosurgery, Uppsala University, Uppsala, Sweden; 3 Department of Clinical Neuroscience, Neuroimmunology Unit, Karolinska Institute, Karolinska University Hospital, Stockholm, Sweden; 4 Department of Neurology and Rehabilitation Medicine, Skåne University Hospital, Lund, Sweden; 5 Department of Health Sciences, Luleå University of Technology, Luleå, Sweden; 6 Department of Clinical Sciences Lund, Neurology, Lund University, Lund, Sweden; 7 Department of Clinical Sciences Lund, Neurosurgery, Lund University, Skåne University Hospital EA-Blocket Plan 4, Klinikgatan 17A7, 221 85 Lund, Sweden

**Keywords:** Neuroinflammation, Biomarkers, Cerebrospinal fluid, Concussion, Sport, Persisting post-concussive symptoms, Cytokines, Chemokines

## Abstract

A sports-related concussion (SRC) is often caused by rapid head rotation at impact, leading to shearing and stretching of axons in the white matter and initiation of secondary inflammatory processes that may exacerbate the initial injury. We hypothesized that athletes with persistent post-concussive symptoms (PPCS) display signs of ongoing neuroinflammation, as reflected by altered profiles of cerebrospinal fluid (CSF) biomarkers, in turn relating to symptom severity. We recruited athletes with PPCS preventing sports participation as well as limiting work, school and/or social activities for ≥ 6 months for symptom rating using the Sport Concussion Assessment Tool, version 5 (SCAT-5) and for cognitive assessment using the Repeatable Battery for the Assessment of Neuropsychological Status (RBANS). Following a spinal tap, we analysed 27 CSF inflammatory biomarkers (pro-inflammatory chemokines and cytokine panels) by a multiplex immunoassay using antibodies as electrochemiluminescent labels to quantify concentrations in PPCS athletes, and in healthy age- and sex-matched controls exercising ≤ 2 times/week at low-to-moderate intensity. Thirty-six subjects were included, 24 athletes with PPCS and 12 controls. The SRC athletes had sustained a median of five concussions, the most recent at a median of 17 months prior to the investigation. CSF cytokines and chemokines levels were significantly increased in eight (IL-2, TNF-α, IL-15, TNF-β, VEGF, Eotaxin, IP-10, and TARC), significantly decreased in one (Eotaxin-3), and unaltered in 16 in SRC athletes when compared to controls, and two were un-detectable. The SRC athletes reported many and severe post-concussive symptoms on SCAT5, and 10 out of 24 athletes performed in the impaired range (*Z* < − 1.5) on cognitive testing. Individual biomarker concentrations did not strongly correlate with symptom rating or cognitive function. Limitations include evaluation at a single post-injury time point in relatively small cohorts, and no control group of concussed athletes without persisting symptoms was included. Based on CSF inflammatory marker profiling we find signs of ongoing neuroinflammation persisting months to years after the last SRC in athletes with persistent post-concussive symptoms. Since an ongoing inflammatory response may exacerbate the brain injury these results encourage studies of treatments targeting the post-injury inflammatory response in sports-related concussion.

## Introduction

A sports-related concussion (SRC) is a mild traumatic brain injury (mTBI) caused by rotational forces transmitted to the brain [1]. At time of impact, rotational as well as linear forces result in shearing and stretching of axons and potentially injury to other components of the white matter. An inflammatory process may accompany these white matter injuries [2], leading to astroglial scar formation, axonal beading, DNA damage, and potentially long-term neurodegeneration [3, 4]. The dynamics of these injury mechanisms remain uncertain, and it has not been established whether they may cease, persist, or even progress with time [5]. Athletes often attain SRCs at increased body temperature from the exercise [6], potentially making their brains vulnerable to secondary injury processes due to an increased metabolic rate from the elevated temperature [7, 8]. Moreover, many athletes sustain several SRCs during their careers. In a majority of athletes, symptoms resolve within 7–14 days following an SRC [9].

However, an increasing number of SRC athletes develop persistent post-concussive symptoms (PPCS) lasting for months to years’ post-injury [10]. These long-lasting symptoms may potentially be a sign of an ongoing, poorly understood, pathologic process in the brain [11]. Neuroinflammation may be a contributing factor to this ongoing injury process, and may be correlated with the functional outcome of athletes following SRC [12].

The aim of this study was to investigate cerebrospinal fluid (CSF) biomarkers of inflammation, and their correlations with cognitive function and post-concussive symptoms in athletes with long-lasting PPCS. If an ongoing inflammatory process could be confirmed, new possibilities for anti-inflammatory treatments in PPCS athletes may emerge.

## Materials and methods

### Ethics

The study was conducted in accordance with the Declaration of Helsinki. All participants received oral and written information, and signed a written consent form prior to participation. The study was approved by the Regional Ethics Committee of Lund University, Lund, Sweden (Dnr 2017/1049), the Regional Ethical Review Board in Stockholm, Sweden (Dnr 2014/1201-31/1), and the Regional Research Ethics Committee in Uppsala, Sweden (Dnr 2015/012).

### Study population

Adult athletes with a history of one or more SRCs and PPCS duration for at least 6 months without symptom resolution at any time were recruited from two locations—the University Hospital in Lund and the University Hospital in Uppsala, Sweden. The PPCS athletes were included it they experienced debilitating symptoms with a severity preventing participation in sports, and limiting work, school, or regular social activities. The 6-month duration was selected in view of the worse prognosis, the longer the duration of symptoms [10, 13]. Moreover, this symptom duration was based on our previous work using tau-PET, where several months post-injury may be required for aggregation of phosphorylated tau following traumatic brain injuries [14]. CSF sampling, the Repeatable Battery for the Assessment of Neuropsychological Status (RBANS), and the Sport Concussion Assessment Tool, version 5 (SCAT5) were analyzed in both cohorts [14].

As controls, CSF samples obtained from healthy individuals, who exercised ≤ 2 times/week and whose exercise habits included physical activities at low-to-moderate intensity, such as power walking or slow jogging and participated in a study at the Karolinska Institute, Stockholm, Sweden of acute effects of aerobic exercise on biomarkers levels in plasma and CSF. The samples analyzed here were obtained at baseline, where participants had been instructed to abstain from any physical exercise 7 days before sampling to limit possible short-term effects of recent exercise on biomarker levels [15].

### SCAT5

SCAT5 [16, 17] contains a graded self-reported symptom checklist, evaluating 22 symptoms in seven rankings on a Likert scale, where 0 is no symptom and 6 is the maximum symptom severity. The total number of symptoms (maximum 22) and symptom severity (maximum 132) are used for presentation of scores.

### RBANS

RBANS is a psychometric test consisting of 12 subtests organised into five index scores and a total global score assessing cognitive function [18]. RBANS was designed for the evaluation of cognitive deficits in neurodegenerative diseases [19] and has been validated for TBI patients [20, 21]. The test was administered by a licensed psychologist.

### CSF sampling

In Lund (*n* = 15), 5 ml of CSF was drawn by a lumbar puncture in a sitting or cumbent side position. An atraumatic needle (22G, 90 mm) was used in the L3–L4, L4–L5 or L5–S1 intervertebral space. Samples were centrifuged at 3000 rpm (corresponding to a relative centrifugal force (RCF) of 1449*g*) at 4 °C in 10 min before pipetted into 1 ml ampullas and stored at − 80 °C within one hour. In Uppsala (*n* = 9), CSF samples were collected by routine lumbar puncture, samples centrifuged at 3450 rpm (*g* 2400) at room temperature for 7 min and stored at—20 °C for 1–2 days following storage at − 80 °C.

CSF from healthy controls were obtained using the same procedure as in Lund, with an atraumatic needle (22G, 70 mm) inserted into the L4–L5 or L5–S1 intervertebral space. A total volume of 25 ml of CSF was collected, with separation of cells by centrifugation for 10 min at room temperature at 350 G, division into aliquots and freezing of supernatant at − 80 °C within 1 h.

The samples from Uppsala and Stockholm were sent to Lund on dried ice and were not thawed prior to or during transport. All samples were then stored at − 80 °C until time of the analysis.

### Analysis of biomarkers

CSF samples were analyzed for inflammatory mediators using the Meso Scale Discovery (MSD; Rockville, MD, USA) MULTISPOT Assay System V-PLEX Human Proinflammatory Panel 1, Cytokine Panel 1, and Chemokine Panel 1. The inflammatory proteins were detected by multiplex immunoassay, using antibodies as electrochemiluminescent labels to quantify concentrations. For each panel, a specific antibody solution containing nine antibodies was used:

*Cytokine Panel 1*: 60 µl containing manufacturer defined SULFO-TAG interleukin (IL)-1 alpha (IL-1α), IL-5, IL-7, IL-12/IL-23p40, IL-15, IL-16, IL-17A, tumor necrosis factor beta (TNF-β), and vascular endothelial growth factor (VEGF) were added to 2400 µl of diluent.

*Proinflammatory Panel 1*: 60 µl containing manufacturer defined SULFO-TAG interferon gamma (IFN-γ), IL-1 beta (IL-1β), IL-2, IL-4, IL-6, IL-8, IL-10, IL-13, and tumor necrosis factor alpha (TNF-α) were added to 2400 µl of diluent.

*Chemokine Panel 1*: 60 µl containing manufacturer defined SULFO-TAG Eotaxin, Eotaxin-3, interferon gamma-induced protein 10 (IP-10), monocyte chemoattractant protein-1 (MCP-1), monocyte chemoattractant protein-4 (MCP-4), macrophage-derived chemokine (MDC), macrophage inflammatory protein-1 alpha (MIP-1α), macrophage inflammatory protein-1 beta (MIP-1β) and thymus- and activation-regulated chemokine (TARC) were added to 2400 µl of diluent.

Undiluted samples were thawed on ice and further equilibrated to room temperature. All steps of the assay were performed according to the manufacturer’s instructions. Plates were washed with 150 µl/well 1 × Wash Buffer (dilution of a 20 × concentrate with deionized water), washing was repeated three times. Sample or calibrator/standard (50 µl/well) were added, plates were sealed, and incubated at room temperature with shaking (1000 rpm) for 2 h. Plates were washed three times. Thereafter, plate specific antibody solutions (25 µl/well) were added, plates sealed and incubated at room temperature with shaking for 2 h. Plates were again washed three times in 1 × Wash buffer. Prior to loading the plate for analysis, a 2 × Read Buffer T (150 µl/well) was added to the plate. Reading and analysis were accomplished on a MESO QuickPlex SQ 120 instrument and the MSD Discovery Workbench software version 4.0.13 (Rockville, MD, USA). All samples were analyzed in duplicates, on the same batch and by the same researcher (AG). Validation of the assays has been performed at the vendor’s site including tests for sensitivity, specificity, accuracy and precision, dynamic range of the calibration curve as well as matrix and samples effects.

### Statistical analysis

The Statistical Package for the Social Sciences (SPSS Inc., Version 28, IBM, New York) was used for all statistical analyzes. For assessment of normality Shapiro–Wilk tests was used. Normally distributed parameters are presented using means and standard deviations (SD) and skewed, nominal or categorical parameters are presented with medians and interquartile ranges (IQR). Log-transformation was not performed [22]. The results of the RBANS are presented as *Z*-scores, based on test-specific published norms [18]. Cognitive impairment is defined by performances $\le$ − 1.5 *Z*, i.e., 1.5 SD below normative means [23].

Because of the relatively small sample size non-parametric tests were used for all comparisons; Mann–Whitney *U* test if the data were nominal or continuous, Chi-Square tests if data were categorical or binominal, and for correlations Spearman’s coefficient (*r*_s_) was calculated.

## Results

### Study population

**Fig. 1:**
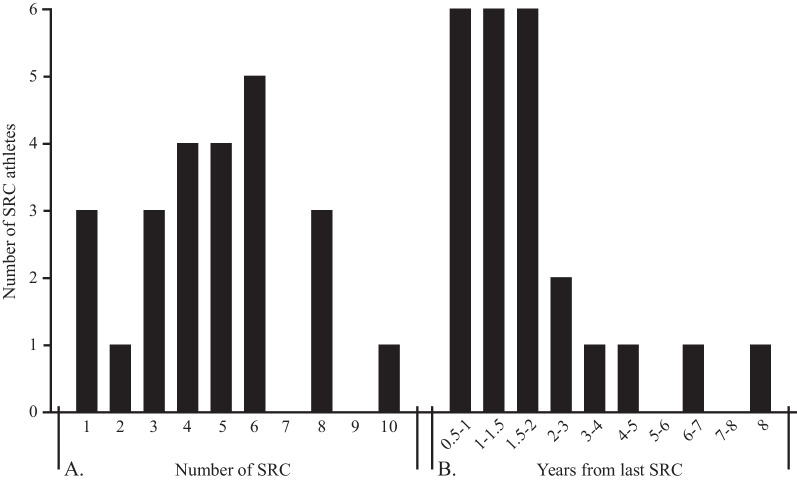
SRC details. **A** Number of sports-related concussions (SRC)s reported by the SRC athletes. **B** Years from last SRC until inclusion in the study amongst the SRC athletes

Thirty-six subjects were included in this study, 24 athletes with previous SRCs and persisting symptoms, and 12 healthy age-, sex- and athletically matched controls. Of the SRC athletes, 15 were recruited in Lund and 9 in Uppsala. There was an equal number of men and women in both groups with the mean age of 26 (range 18–36) years, without differences among the groups (*p* > 0.05). The SRC athletes had sustained a median of 5 (IQR 1–10) concussions, at a median of 17 (IQR 10.5–26) months since the last SRC (Fig. 1). Demographics and SRC details are listed in Table 1. No athlete, or control, were active smokers, and three (out of 24) PPCS athletes were on antidepressants at time of CSF sampling.

**Table 1:** Subject characteristics

	SRC athletes, *n* = 24	Controls, *n* = 12
Male sex, % (*n*),* p* = 0.486	54% (13)	42%, (5)
Age, mean (SD),* p* = 0.103	25 (5.1) years	28 (4.9) years
Sports (*n*)	Ice hockey (9), soccer (5), karate (4), alpine skiing (2), indoor hockey (2), handball (1), and wrestling (1)	Running (12)
Years of practice in the contact sport, mean (SD)*	17.8 (5.0)	
Number of SRCs, mean (range)	5 (1–10)	–
Time from last SRC, median (IQR)	17 (10.5–26) months	–
SCAT5 number of symptoms, median (IQR)	19 (14–22)	–
SCAT5 symptom severity, median (IQR)	51 (IQR 33–74)	–
RBANS, *Z*-score (SD)	− 1.25 (1.20)	–

The Sport Concussion Assessment Tool 5h edition (SCAT5) has a maximum symptom severity of 132 and a maximum number of symptoms of 22

*SD*  standard deviation and *IQR* interquartile range

*Data from the Lund cohort

The SRC athletes reported a median of 19 (IQR 14–22) symptoms and a median symptom severity score of 51 (IQR 33–74) on SCAT5. Cognitive function measured with RBANS global score was impaired in 10 out of the 24 athletes. Thus, the proportion of SRC athletes with impaired performance below *Z* < − 1.5 was 6 times more common than would be expected in an age-related normal population (42% vs. 7%). However, when comparing SRC athletes as a group with published norm data [18] a significant difference was not obtained. There were no differences between male and female athletes in number of SRCs, RBANS, SCAT5 symptom severity scores and number of symptoms.

### Biomarkers

Biomarkers are presented, based on their skewed distribution, as medians and IQR in Table 2.

**Table 2:** Biomarkers

	Biomarkers, pg/ml	SRC athletes, *n* = 24 median (IQR)	Controls, *n* = 12 median (IQR)	*P*-value	Duplicate cv (%)	LLOD	No. samples below detection
Cytokine Panel 1	IL-5	8.44 (7.19–9.73)	8.26 (6.84–9.41)	0.737	2.83	2.00	0
	IL-7	9.82 (7.32–12.8)	10.4 (7.62–14.0)	0.663	4.64	1.06	0
	IL-12/IL-23p40	16.7 (14.2–21.8)	13.8 (11.7–17.2)	0.052	3.83	1.61	0
	**IL-15**	**44.4 (39.3–53.2)**	**37.1 (34.5–44.7)**	**0.025**	4.14	2.01	0
	IL-16	75.8 (46.8–128)	57.2 (49.3–60.7)	0.107	4.56	8.47	0
	IL-17A	0.51 (0.37–0.62)	0.47 (0.37–0.51)	0.330	6.71	1.00	1
	**TNF-β**	**1.05 (0.80–1.37)**	**0.48 (0.08–0.73)**	**0.002**	4.29	1.94	5
	**VEGF**	**37.7 (31.9–40.9)**	**27.6 (24.4–32.5)**	**0.012**	3.97	18.90	0
Proinflammatory panel 1	IFN-γ	7.37 (5.55–9.25)	7.12 (4.91–8.68)	0.867	4.41	4.15	0
	IL-1β	1.65 (0.64–2.77)	0.80 (0.00–3.33)	0.420	6.53	2.39	6
	**IL-2**	**0.98 (0.77–1.20)**	**0.75 (0.64–0.90)**	**0.032**	4.81	0.96	0
	IL-6	21.2 (13.4–28.3)	21.9 (17.9–29.4)	0.591	6.44	1.59	0
	IL-8	904 (644–1034)	676 (625–841)	0.093	8.76	1.60	0
	IL-10	2.61 (2.11–3.49)	2.17 (1.69–2.52)	0.087	5.91	1.72	0
	IL-13	51.5 (44.6–59.9)	48.8 (39.6–54.2)	0.247	9.21	51.4	2
	**TNF-α**	**8.80 (6.19–9.99)**	**6.35 (5.00–6.94)**	**0.011**	5.39	4.87	0
Chemokine Panel 1	**Eotaxin**	**67.0 (55.8–81.2)**	**49.8 (44.2–64.5)**	**0.008**	11.66	24.00	0
	**Eotaxin-3**	**5.81 (4.48–8.59)**	**8.90 (7.99–14.3)**	**0.006**	12.43	2.86	0
	**IP-10**	**1198 (905–2224)**	**570 (499–1249)**	**0.019**	12.65	0.94	0
	MCP-1	6178 (5065–6811)	6108 (5675–8900)	0.383	6.15	1.35	0
	MCP-4	62.7 (47.3–81.3)	56.82 (50.1–72.1)	0.421	9.04	31.00	1
	MDC	31.8 (25.7–37.9)	30.3 (25.7–33.5)	0.460	6.40	7.42	0
	MIP-1α	78.9 (74.2–87.8)	80.1 (72.1–83.8)	0.867	7.64	27.90	0
	MIP-1β	89.7 (61–115)	81.8 (63.6–125)	0.737	9.68	6.31	0
	**TARC**	**37.2 (29.7–53.3)**	**26.8 (25.1–40.7)**	**0.027**	10.62	1.38	0

Analyzed biomarkers in cerebrospinal fluid in athletes with SRC and healthy controls were: Interleukin (IL)-1 alpha (IL-1α), IL-5, IL-7, IL-12/IL-23p40, IL-15, IL-16, IL-17A, tumor necrosis factor beta (TNF-β), vascular endothelial growth factor (VEGF), interferon gamma (IFN-γ), IL-1 beta (IL-1β), IL-2, IL-4, IL-6, IL-8, IL-10, IL-13, tumor necrosis factor alpha (TNF-α), Eotaxin, Eotaxin-3, interferon gamma-induced protein 10 (IP-10), monocyte chemoattractant protein-1 (MCP-1), monocyte chemoattractant protein-4 (MCP-4), macrophage-derived chemokine (MDC), macrophage inflammatory protein-1 alpha (MIP-1α), macrophage inflammatory protein-1 beta (MIP-1β) and thymus- and activation-regulated chemokine (TARC). The biomarkers are presented as medians and interquartile ranges (IQR). *LLOD*  lower limit of detection, *CV*  coefficient of variation, *No*  number. Statistically significant differences were defined as a *P*-value < 0.05 and are bolded

Of the 27 tested biomarkers, the cytokines IL-4 and IL-1α levels were non-detectable, or lower than the limit of detection suggested by the manufacturer, in 21 and 30 of the 36 analyzed subjects, respectively, and were, therefore, excluded. Thus, 25 inflammatory markers were, therefore, included for further analyses.

**Fig. 2:**
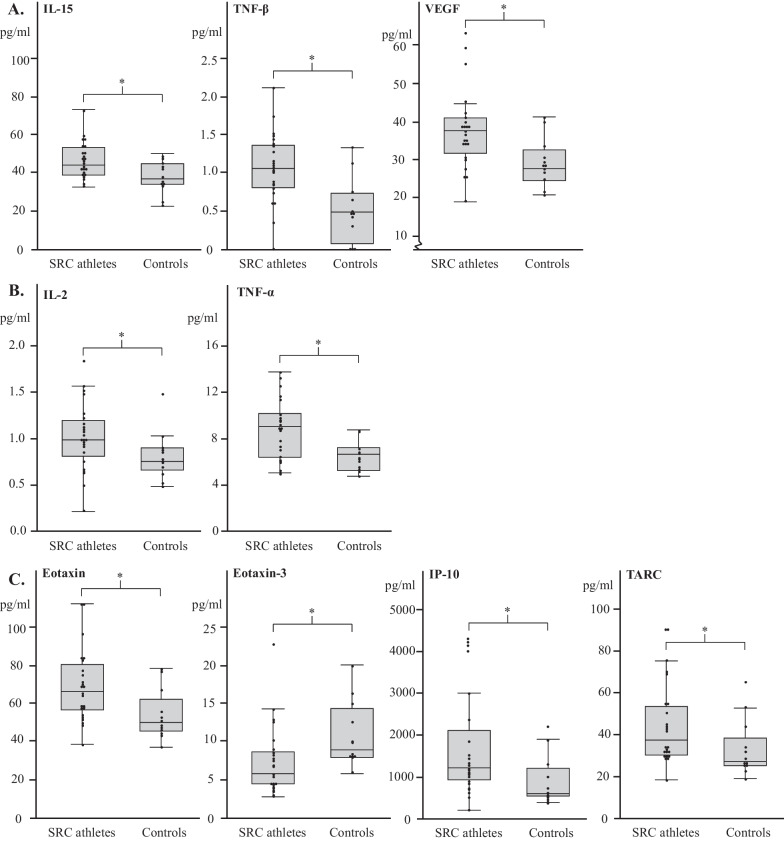
Cerebrospinal fluid biomarkers of neuroinflammation are predominately increased in athletes with persistent post-concussive symptoms. **A** Cytokine panel 1: Interleukin (IL)-5, tumor necrosis factor beta (TNF-β), and vascular endothelial growth factor (VEGF). **B** Proinflammatory panel 1: IL-2 and tumor necrosis factor alpha (TNF-α). **C** Chemokine panel 1: Eotaxin, Eotaxin-3, interferon gamma-induced protein 10 (IP-10), and thymus- and activation-regulated chemokine (TARC). Biomarkers are presented as boxplots in pg/ml and individual levels indicated by dots. *Indicates a significant difference (*p* < 0.05) in the evaluated biomarker between concussed athletes and athletic age- and sex matched controls

Of the 25 included biomarkers of inflammation, in general increased levels were found in SRC athletes. Of the included biomarkers, 8 were significantly higher in SRC athletes than in controls (IL-2; *p* = 0.032, TNF-α; *p* = 0.011, IL-15; *p* = 0.025, TNF-β; *p* = 0.002, VEGF; *p* = 0.012, Eotaxin; *p* = 0.008, IP-10*; p* = 0.019, and TARC; *p* = 0.027, Fig. 2).

The CSF level of one biomarker (Eotaxin-3) was significantly lower in SRC athletes than in controls (*p* = 0.006, Fig. 2).

### Correlations

RBANS global score correlated negatively with MIP-1α (*r*_s_ = − 0.432, *p* = 0.035). SCAT5 number of symptoms correlated negatively with Eotaxin-3 (*r*_s_ = − 0.512, *p* = 0.013) and TARC (*r*_s_ = − 0.428, *p* = 0.041). SCAT5 symptom severity score correlated negatively with IP-10 (*r*_s_ = − 0.413, *p* = 0.050) No other biomarker correlated with RBANS or SCAT5 number of symptoms and symptom severity score.

## Discussion

The present study explored the profile of CSF inflammatory biomarkers in athletes with post-concussive symptoms persisting more than 6 months following their most recent sports-related concussion (SRC). Compared to age- and sex-matched athletic controls, we identified increased levels in eight out of the 25 inflammatory biomarkers. This implies an ongoing inflammatory process in the brain, as reflected in the CSF, several months or years following the injury that potentially contributes to the persistent symptoms.

Axonal stretching and shearing may occur at time of the head impact that results in an SRC, accompanied by a neuroinflammatory cascade that is triggered within minutes post-injury. This inflammatory response can continue for several months to years, and lead to a chronic inflammatory phase that may be maladaptive and exacerbate the initial injury [24]. These long-lasting inflammatory processes may then lead to secondary brain injury, reactive pathological processes such as astrogliosis, microglial activation, axonal beading, DNA damage and neurodegeneration [3, 4, 25–27].

The inflammatory response that occurs following an SRC appears less marked, and more characterized by gliosis [24], than that observed in severe TBI [14, 28]. The neuroinflammatory cascade after TBI is complex and involves the activation of resident microglia, increased permeability of the blood–brain barrier, recruitment of peripheral immune cells, tissue damage and cytokine release [29]. While direct evidence of an inflammatory response in humans is rare, when four young athletes who died from other causes shortly after SRCs were investigated clusters of activated perivascular microglia were observed in subcortical white matter tracts indicating an inflammatory response [30].

In a mouse model of mild TBI (mTBI), an early increase in serum and brain cytokines, associated with an impaired motor function, was observed [31, 32]. One single mTBI in mice was found to elicit parenchymal neuroinflammation in the cortex and hippocampus up to three weeks post-injury on histology and micro-PET imaging [33]. In both single and repeated mTBI, mimicking SRC, axonal injury and neuroinflammation play significant roles in the neuropathological events that include ongoing white matter degradation up to 12 month post-injury, and while direct causality remains to be proven the inflammatory response is associated with chronic functional impairments [34, 35]. The neuroinflammatory response also appears exacerbated by repeated mTBIs in the experimental setting [34, 36], and may be attenuated by treatment approaches [37].

In mTBI patients, there is a distinct inflammatory signature that correlates with functional and cognitive outcome [2, 38], and inflammatory markers may predict recovery following the brain injury [39]. A large longitudinal study showed that mTBI patients, injured outside of a sports context, had a prolonged increase in serum cytokines for 1 year post-injury, reflecting a low-level and persistent systemic inflammation following mTBI [40]. These studies were, however, conducted on peripheral blood samples and may thus partly represent also a systemic inflammatory response [15]. Blood samples were only available in a subset of our PPCS athletes, and could not be used for comparison with the CSF cytokine levels obtained in the present study. In fact, plasma cytokine levels may poorly reflect the intracerebral inflammation, with large differences between brain parenchymal and systemic cytokine concentrations, as shown in severe TBI [41] or intracerebral hemorrhage [42].

To date, and to the best of our knowledge, there are no previous studies investigating inflammatory mediators in the CSF of SRC athletes in the chronic phase.

CSF sampling is invasive and not entirely without risks, and other indirect methods have included neuroimaging studies, such as PET. PET imaging using a tracer binding to translocator proteins can be used as a marker of microglial activation and astrocytosis. In former professional American football players, increased neuroinflammation and hippocampal atrophy was observed 24–42 years after retirement [43], and at a mean of 7 years following the most recent SRC [44]. Moreover, glial fibrillary acidic protein (GFAP), a biomarker of astrogliosis [45], was found to increase acutely and remain elevated several years following SRC [46, 47]. Finally, we recently observed by PET imaging increased neuroinflammation in the hippocampus of young SRC athletes > 6 months following their last SRC [14], of relevance in view of the observed cognitive impairment observed here. Taken together, these studies and our present work find evidence of both an acute and chronic neuroinflammatory process triggered by SRC. Since there is much evidence linking axonal injury, neuroinflammation and neurodegeneration [48–50], the persistent neuroinflammation observed in our present study may be detrimental.

The immune response following TBI is complex and time dependent, and it involves both pro- and anti-inflammatory mechanisms [24]. While cytokines are often classified as either pro- or anti-inflammatory, this may be an oversimplification, since many cytokines can have a dual role determined by the activation signal, the timing, and the target cell [51]. The levels of cytokines vary also depending on the analyzed timepoint following TBI [2, 31, 40, 41], suggesting that the temporal aspect is of importance in the interpretation of these results. In the present study, we observed a significant elevation of many cytokines in the CSF of SRC athletes at a mean of 17 month post-injury, of which several may have both pro- and anti-inflammatory roles. However, based on the evidence presented in the previous paragraphs, it is plausible that the increased neuroinflammation exacerbates the brain injury in chronic SRC.

We did not find any convincing correlations between cytokines and neither the cognitive impairment nor the post-concussion symptoms, which may be explained by the homogeneity of the included SRC athletes. Most of the SRC athletes reported many and severe post-concussion symptoms and scored low on cognitive function, with a low dispersal within the group. Inclusion of asymptomatic SRC athletes may have altered these results. Previously a correlation between blood cytokines and post-concussion symptoms, depression, and post-traumatic stress were found [2]. Although the time course of inflammation, functional impairment and symptoms has not been established, a PET-imaging study of American football players suggested that neuroinflammation may precede cognitive symptoms [44]. Our cohort of athletes had persisting symptoms since their last SRC, without a symptom free period, and these symptoms may be associated with an ongoing neuroinflammation. To establish this association, further studies including athletes with diverging symptom burden, including those who recover fully from SRC-induced symptoms, and a longitudinal follow-up design is warranted. In previous studies, inflammatory biomarker profiles are different in athletes with a concussion history when compared to athletes with no previous SRC from the same sport [52] and be related to neuroimaging findings [53]. There also seem to be sex differences in several biomarker studies on inflammation [52, 54], findings that need confirmation in larger studies. Although all included athletes were without abnormalities on routine neuroimaging, our PPCS cohort had a markedly high symptom burden as assessed by the SCAT-5. This, our findings may not be similar in less symptomatic SRC athletes. Moreover, our athletes had long careers in their contact sports and the cumulative effects on the inflammatory biomarkers remain undetermined.

Our present study has limitations. We aimed for including young athletes with long-term persisting symptoms following SRC, thus this selected cohort may not be representative of asymptomatic SRC athletes. This presumably influenced the correlation analyses, where a more diverged group may have produced other results. Biomarkers of inflammation was analyzed at one time-point, and therefore, the temporal dynamics of the inflammatory process following SRC was not established. In addition, there were minor methodological differences in the processing of the CSF samples among the study sites that may have affected some analyses. Most of our athletes, with one exception, also had previous SRCs throughout their career and we cannot establish whether the observed increase in the inflammatory markers were caused by their most recent SRC, or a cumulative effect of the previous SRCs. The sample size is rather small, reflected by the strict inclusion criteria used here. We also used athletic controls (runners) without any previous SRC, and we cannot exclude that their inflammatory biomarker profile is different from non-athletic controls. In view of their symptom severity, the basal activity of the included PPCS athletes was presumably low. While our control cohort did not exercise the week prior to CSF sampling, we cannot exclude that their activity level, in general, was higher that the included PPCS athletes. However, it should be noted that even after acute strenuous activity, the changes in CSF cytokines are rather modest [15] and the influence of general activity on our present biomarker results is presumably low. Finally, this was an explorative study analyzing many inflammatory markers in a relatively small cohort. To avoid the risk of type II errors, multiple comparisons were not performed [55]. However, with an alpha set to 0.05, 10% of tests would be expected to be false positives. Thus, if more than 10% of analyses were different between groups at the 0.05 level, it is unlikely that this was explained merely by statistical chance. Despite the difficulties recruiting for CSF sampling, a study using a larger cohort of concussed athletes may be needed to confirm the present results.

## Conclusions

In this first study, examining CSF biomarkers of neuroinflammation in SRC athletes with long-term persisting post-concussion symptoms, at a median of 17 month post-injury, we present evidence of an ongoing neuroinflammation. Eight inflammatory mediators were found to be chronically elevated. Furthermore, the SRC athletes report many and severe post-concussion symptoms and signs of cognitive impairment, to which the neuroinflammation may have contributed. Our results support the view of SRC is a chronic disorder complicated by a persistent inflammatory process, which may provide a novel target for anti-inflammatory therapies.

## Data Availability

The data sets used and/or analysed during the current study are available from the corresponding author on reasonable request.

## Abbreviations

SRC: Sport related concussion
TBI: Traumatic brain injury
mTBI: Mild traumatic brain injury
CSF: Cerebrospinal fluid
PPCS: Persistent post-concussive symptoms
SCAT-5: Sport Concussion Assessment Tool, version 5
RBANS: Repeatable Battery for the Assessment of Neuropsychological Status
ELISA: Enzyme-linked immunosorbent assay
PET: Positron-emission tomography
IL: Interleukin
TNF: Tumor necrosis factor
VEGF: Vascular endothelial growth factor
IFN: Interferon
IP: Interferon gamma-induced protein
MCP: Monocyte chemoattractant protein
MDC: Macrophage-derived chemokine
MIP: Macrophage inflammatory protein
TARC: Thymus- and activation-regulated chemokine

## References

[1] McCrory P, Feddermann-Demont N, Dvorak J (2017). What is the definition of sports-related concussion: a systematic review. Br J Sports Med.

[2] Vedantam A, Brennan J, Levin HS (2021). Early versus late profiles of inflammatory cytokines after mild traumatic brain injury and their association with neuropsychological outcomes. J Neurotrauma.

[3] Schwab N, Grenier K, Hazrati LN (2019). DNA repair deficiency and senescence in concussed professional athletes involved in contact sports. Acta Neuropathol Commun.

[4] Tang-Schomer MD, Johnson VE, Baas PW, Stewart W, Smith DH (2012). Partial interruption of axonal transport due to microtubule breakage accounts for the formation of periodic varicosities after traumatic axonal injury. Exp Neurol.

[5] Patterson ZR, Holahan MR (2012). Understanding the neuroinflammatory response following concussion to develop treatment strategies. Front Cell Neurosci.

[6] Batchelder BC, Krause BA, Seegmiller JG, Starkey CA (2010). Gastrointestinal temperature increases and hypohydration exists after collegiate men's ice hockey participation. J Strength Cond Res.

[7] Sakurai A, Atkins CM, Alonso OF, Bramlett HM, Dietrich WD (2012). Mild hyperthermia worsens the neuropathological damage associated with mild traumatic brain injury in rats. J Neurotrauma.

[8] Bonds BW, Hu P, Li Y (2015). Predictive value of hyperthermia and intracranial hypertension on neurological outcomes in patients with severe traumatic brain injury. Brain Inj.

[9] Tator CH, Davis HS, Dufort PA (2016). Postconcussion syndrome: demographics and predictors in 221 patients. J Neurosurg.

[10] Hiploylee C, Dufort PA, Davis HS (2017). Longitudinal study of postconcussion syndrome: not everyone recovers. J Neurotrauma.

[11] Shahim P, Tegner Y, Gustafsson B (2016). Neurochemical aftermath of repetitive mild traumatic brain injury. JAMA Neurol.

[12] Shahim P, Zetterberg H (2022). Neurochemical markers of traumatic brain injury: relevance to acute diagnostics, disease monitoring, and neuropsychiatric outcome prediction. Biol Psychiatry.

[13] Johnson VE, Stewart W, Smith DH (2012). Widespread tau and amyloid-beta pathology many years after a single traumatic brain injury in humans. Brain Pathol.

[14] Marklund N, Vedung F, Lubberink M (2021). Tau aggregation and increased neuroinflammation in athletes after sports-related concussions and in traumatic brain injury patients—a PET/MR study. Neuroimage Clin.

[15] Isung J, Granqvist M, Trepci A (2021). Differential effects on blood and cerebrospinal fluid immune protein markers and kynurenine pathway metabolites from aerobic physical exercise in healthy subjects. Sci Rep.

[16] Sport concussion assessment tool, 5th edn. Br J Sports Med. 2017;51:851–8. 10.1136/bjsports-2017-097506SCAT5 28446451

[17] Echemendia RJ, Meeuwisse W, McCrory P (2017). The Sport Concussion Assessment Tool 5th Edition (SCAT5): background and rationale. Br J Sports Med.

[18] Randolph C. Repeatable battery for the assessment of neuropsychological status—RBANS. Stockholm: Pearson Assessment; 2013.

[19] Randolph C, Tierney MC, Mohr E, Chase TN (1998). The Repeatable Battery for the Assessment of Neuropsychological Status (RBANS): preliminary clinical validity. J Clin Exp Neuropsychol.

[20] McKay C, Casey JE, Wertheimer J, Fichtenberg NL (2007). Reliability and validity of the RBANS in a traumatic brain injured sample. Arch Clin Neuropsychol.

[21] Pachet AK (2007). Construct validity of the Repeatable Battery of Neuropsychological Status (RBANS) with acquired brain injury patients. Clin Neuropsychol.

[22] Feng C, Wang H, Lu N (2014). Log-transformation and its implications for data analysis. Shanghai Arch Psychiatry.

[23] Petersen RC, Morris JC (2005). Mild cognitive impairment as a clinical entity and treatment target. Arch Neurol.

[24] Jassam YN, Izzy S, Whalen M, McGavern DB, El Khoury J (2017). Neuroimmunology of traumatic brain injury: time for a paradigm shift. Neuron.

[25] Engel S, Schluesener H, Mittelbronn M (2000). Dynamics of microglial activation after human traumatic brain injury are revealed by delayed expression of macrophage-related proteins MRP8 and MRP14. Acta Neuropathol.

[26] Witcher KG, Bray CE, Chunchai T (2021). Traumatic brain injury causes chronic cortical inflammation and neuronal dysfunction mediated by microglia. J Neurosci.

[27] Johnson VE, Stewart JE, Begbie FD (2013). Inflammation and white matter degeneration persist for years after a single traumatic brain injury. Brain.

[28] Ramlackhansingh AF, Brooks DJ, Greenwood RJ (2011). Inflammation after trauma: microglial activation and traumatic brain injury. Ann Neurol.

[29] Jacquens A, Needham EJ, Zanier ER, Degos V, Gressens P, Menon D (2022). Neuro-inflammation modulation and post-traumatic brain injury lesions: from bench to bed-side. Int J Mol Sci.

[30] Goldstein LE, Fisher AM, Tagge CA (2012). Chronic traumatic encephalopathy in blast-exposed military veterans and a blast neurotrauma mouse model. Sci Transl Med.

[31] Yang SH, Gustafson J, Gangidine M (2013). A murine model of mild traumatic brain injury exhibiting cognitive and motor deficits. J Surg Res.

[32] Yang SH, Gangidine M, Pritts TA, Goodman MD, Lentsch AB (2013). Interleukin 6 mediates neuroinflammation and motor coordination deficits after mild traumatic brain injury and brief hypoxia in mice. Shock.

[33] Drieu A, Lanquetin A, Prunotto P (2022). Persistent neuroinflammation and behavioural deficits after single mild traumatic brain injury. J Cereb Blood Flow Metab.

[34] Mouzon BC, Bachmeier C, Ferro A (2014). Chronic neuropathological and neurobehavioral changes in a repetitive mild traumatic brain injury model. Ann Neurol.

[35] Mouzon BC, Bachmeier C, Ojo JO (2018). Lifelong behavioral and neuropathological consequences of repetitive mild traumatic brain injury. Ann Clin Transl Neurol.

[36] Shultz SR, Bao F, Omana V (2012). Repeated mild lateral fluid percussion brain injury in the rat causes cumulative long-term behavioral impairments, neuroinflammation, and cortical loss in an animal model of repeated concussion. J Neurotrauma.

[37] Webster KM, Wright DK, Sun M (2015). Progesterone treatment reduces neuroinflammation, oxidative stress and brain damage and improves long-term outcomes in a rat model of repeated mild traumatic brain injury. J Neuroinflamm.

[38] Huie JR, Diaz-Arrastia R, Yue JK (2019). Testing a multivariate proteomic panel for traumatic brain injury biomarker discovery: a TRACK-TBI pilot study. J Neurotrauma.

[39] Meier TB, Huber DL, Bohorquez-Montoya L (2020). A prospective study of acute blood-based biomarkers for sport-related concussion. Ann Neurol.

[40] Chaban V, Clarke GJB, Skandsen T (2020). Systemic inflammation persists the first year after mild traumatic brain injury: results from the prospective trondheim mild traumatic brain injury study. J Neurotrauma.

[41] Helmy A, Carpenter KL, Menon DK, Pickard JD, Hutchinson PJ (2011). The cytokine response to human traumatic brain injury: temporal profiles and evidence for cerebral parenchymal production. J Cereb Blood Flow Metab.

[42] Tobieson L, Gard A, Ruscher K, Marklund N (2022). Intracerebral proinflammatory cytokine increase in surgically evacuated intracerebral hemorrhage: a microdialysis study. Neurocrit Care.

[43] Coughlin JM, Wang Y, Munro CA (2015). Neuroinflammation and brain atrophy in former NFL players: an in vivo multimodal imaging pilot study. Neurobiol Dis.

[44] Coughlin JM, Wang Y, Minn I (2017). Imaging of glial cell activation and white matter integrity in brains of active and recently retired national football league players. JAMA Neurol.

[45] Bignami A, Eng LF, Dahl D, Uyeda CT (1972). Localization of the glial fibrillary acidic protein in astrocytes by immunofluorescence. Brain Res.

[46] McCrea M, Broglio SP, McAllister TW (2020). Association of blood biomarkers with acute sport-related concussion in collegiate athletes: findings from the NCAA and Department of Defense CARE Consortium. JAMA Netw Open.

[47] Shahim P, Politis A, van der Merwe A (2020). Time course and diagnostic utility of NfL, tau, GFAP, and UCH-L1 in subacute and chronic TBI. Neurology.

[48] Cherry JD, Tripodis Y, Alvarez VE (2016). Microglial neuroinflammation contributes to tau accumulation in chronic traumatic encephalopathy. Acta Neuropathol Commun.

[49] Collins-Praino LE, Arulsamy A, Katharesan V, Corrigan F (2018). The effect of an acute systemic inflammatory insult on the chronic effects of a single mild traumatic brain injury. Behav Brain Res.

[50] Holleran L, Kim JH, Gangolli M (2017). Axonal disruption in white matter underlying cortical sulcus tau pathology in chronic traumatic encephalopathy. Acta Neuropathol.

[51] Cavaillon JM (2001). Pro- versus anti-inflammatory cytokines: myth or reality. Cell Mol Biol (Noisy-le-grand).

[52] Di Battista AP, Rhind SG, Richards D (2016). Altered blood biomarker profiles in athletes with a history of repetitive head impacts. PLoS ONE.

[53] Di Battista AP, Churchill N, Schweizer TA (2018). Blood biomarkers are associated with brain function and blood flow following sport concussion. J Neuroimmunol.

[54] O'Brien WT, Symons GF, Bain J (2021). Elevated serum interleukin-1beta levels in male, but not female, collision sport athletes with a concussion history. J Neurotrauma.

[55] Perneger TV (1998). What's wrong with Bonferroni adjustments. BMJ.

